# The study of degradation mechanisms of glyco-engineered plant produced anti-rabies monoclonal antibodies E559 and 62-71-3

**DOI:** 10.1371/journal.pone.0209373

**Published:** 2018-12-20

**Authors:** Sindisiwe G. Buthelezi, Heini W. Dirr, Ereck Chakauya, Rachel Chikwamba, Lennart Martens, Tsepo L. Tsekoa, Elien Vandermarliere, Stoyan H. Stoychev

**Affiliations:** 1 Council for Scientific and Industrial Research, Biosciences Unit, Pretoria, South Africa; 2 Protein Structure-Function Research Unit, School of Molecular and Cell Biology, University of the Witwatersrand, Johannesburg, South Africa; 3 Unit for Computational Omics and Systems Biology, VIB-UGent Center for Medical Biotechnology, VIB, Ghent, Belgium; 4 Department of Biochemistry, Faculty of Medicine and Health Sciences, Ghent University, Ghent, Belgium; University of Texas, UNITED STATES

## Abstract

Rabies is an ancient and neglected zoonotic disease caused by the rabies virus, a neurotropic RNA virus that belongs to the *Rhabdoviridae* family, genus *Lyssavirus*. It remains an important public health problem as there are cost and health concerns imposed by the current human post exposure prophylaxis therapy. The use of monoclonal antibodies (mAbs) is therefore an attractive alternative. Rabies mostly affects people that reside in resource-limited areas where there are occasional failures in the cold-chain. These environmental changes may upset the stability of the mAbs. This study focused on mAbs 62-71-3 and E559; their structures, responses to freeze/thaw (F/T) and exposure to reactive oxygen species were therefore studied with the aid of a wide range of biophysical and *in silico* techniques in order to elucidate their stability and identify aggregation prone regions. E559 was found to be less stable than 62-71-3. The complementarity determining regions (CDR) contributed the most to its instability, more specifically: peptides ^99^EIWD^102^ and ^92^ATSPYT^97^ found in CDR3, Trp33 found in CDR1 and the oxidised Met34. The constant region “^158^SWNSGALTGHTFPAVL^175^” was also flagged by the special aggregation propensity (SAP) tool and F/T experiments to be highly prone to aggregation. The E559 peptides “^4^LQESGSVL^11^ from the heavy chain and ^4^LTQSPSSL^11^ from the light chain, were also highly affected by F/T. These residues may serve as good candidates for mutation, in the aim to bring forward more stable therapeutic antibodies, thus paving a way to a more safe and efficacious antibody-based cocktail treatment against rabies.

## Introduction

Developing countries of Africa and Asia remain highly affected by rabies which is one of the oldest recorded infections of mankind. Rabies is caused by a rod-shaped virus–the rabies virus–that belongs to the *Rhabdovirus* family [1,2]. Its ability to infect several mammalian carnivores and chiroptera species has protected it from total eradication [3]. Nowadays, human infections are mainly due to a bite from a rabid dog [1]. Fatalities can be prevented by thoroughly cleaning the site of injury shortly after the presumed exposure to the virus [4]. This should be promptly followed by post exposure prophylaxis (PEP). Modern PEP protocols include passive antibody therapy (rabies immunoglobulin, RIG) for virus neutralization at the wound site and are followed by active immunisation using the rabies vaccine. There are however various challenges with the current human PEP such as availability, affordability and safety. This is mainly because RIG is prepared from pooled sera from hyper immunised humans (HRIG) or horses (ERIG) [5]. These challenges have therefore motivated several researchers to identify alternative treatments [6,7].

The high specificity and potency of therapeutic monoclonal antibodies (mAbs) and their clinical and commercial successes have made them an attractive alternative to RIG [4,8]. In previous work [9], we discussed the use of E559 and 62-71-3 in a cocktail as each mAb targets a different site of the rabies virus glycoprotein and as such prevents viral escape [4,6]. These mAbs were expressed in ΔxT/FT plants, a *Nicotiana benthamiana* mutant that supports production of fructose-free glycans, and were reported to be more efficacious then RIG. Expression levels attained in the transient system were higher than transgenic approaches which makes this system a suitable basis for an economically viable manufacturing process [9]. Efficacy studies indicated 62-72-3 to be most efficacious, followed by E559 and HRIG [9]. This also indicated that a cocktail of 62-71-3 and E559 could be a good replacement for the current commercially available HRIG.

However, the use of these biological macro-molecules as therapeutic agents comes with its own challenges, as they are highly susceptible to physical and mechanical degradation pathways. Aggregation has been identified as the most relevant physical degradation pathway as it leads to a decrease in efficacy [10]. Moreover, administration of aggregated immunoglobulins can be fatal or lead to side-effects such as renal failure and anaphylactic reactions [11]. Accelerated thermal stability studies were also conducted in our previous study (9) by exposing the mAbs to temperatures that range from 25°C to 90°C, to determine the extent of heat-induced denaturation. Differences were observed from 50–55°C indicating possible rearrangement in secondary structural content in the case of E559. On the other hand, changes in β-sheet content, for 62-71-3, were only observed above 65°C therefore indicating that E559 is less thermostable than 62–71–3.

Oxidation is one of the most common chemical degradation pathways. It can also lead to a decrease in efficacy if it occurs in the complementary determining regions (CDRs) of the mAbs [12,13]. Moreover, it may also lead to aggregation by the creation of new sticky patches on the protein surface or through production of charge heterogeneity that may reduce electrostatic repulsion between monomers which eventually leads to aggregation [14].

The skewed disease burden towards poor rural communities provides an additional challenge as these mAbs would have to be delivered to remote areas thus facing cold-chain challenges [10]. In this study, the E559 and 62-71-3 mAb structures were studied in combination with their responses to freeze/thaw (F/T) and exposure to reactive oxygen species, to understand the mechanisms behind their degradation and to suggest ways to improve their stability.

## Materials and methods

### Chemicals

All chemicals were purchased from Sigma-Aldrich (St. Louis, USA) unless otherwise stated.

### *In silico* structural analysis

#### Homology modelling

The fragment crystallisable (Fc) regions of the chimeric E559 and 62-71-3 were identical and therefore modelled with the same template. Experimentally solved structure templates for the heavy and light chains were identified with the aid of PSI-BLAST [15] which uses a non-redundant database (at 95% redundancy) of structures in the PDB [16]. The variable light (V_L_) and variable heavy (V_H_) chain were searched independently in an in-house germline database to identify templates from PDB with the highest degree of similarity to the CDR regions. The templates used for E559 Fab were; HC template: PDB-entry 1RJL [17]; LC template: PDB-entry 2VXU [18], for 62-71-3 FAB they were; HC template: PDB-entry 1FDL [19]; LC template: PDB-entry 2A6I [20] and template: PDB-entry 1L6X [21] was used for the Fc region.

Next, the sequences of the mAbs of interest were aligned with the candidate templates using ALIGN123 which is available in Accelrys Discovery Studio version 4 [22]. Homology models of the Fab and Fc regions of the mAbs were then built by using MODELER [23], which is also available in Accelrys Discovery Studio version 4. The side-chains of all the residues were refined to optimise their conformation. The modelled fragments were joined using the structure superimposing method of Accelrys Discovery Studio version 4 [22]. Finally, the quality of the modelled structures was evaluated with both PROCHECK version 3.6.2 and WHAT-CHECK [24,25].

#### Spatial aggregation propensity

Aggregation-prone regions were identified by using the spatial aggregation propensity (SAP) as described by Chennamsetty [26]. SAP calculates the hydrophobicity of the dynamically exposed residues found on the protein surface. This algorithm is based on the equation below [26]:

$$\left(SAP)atom\;i=\underset{simulation\;av}{\sum }\{\underset{residue\;with\;at\;least\;one\;side\;chain\;atom\;with\;R\;from\;atom\;i}{\sum }(\frac{SAA\;of\;side\;chain\;atoms\;within\;radius\;R}{SAA\;of\;side\;chain\;atom\;of\;fully\;exposed\;residue})X\;residue\;hydrohobicity\right\}$$

The equation was explained by Chennamsetty and colleagues [26] as: 1) the solvent accessible area (SAA) of the side chain was computed within radius (R = 10 Å) from a given atom. 2) The SAA of a side chain of a fully exposed residue (e.g., for amino acid X) was obtained by calculating the SAA of side chains of the middle residue in the fully extended conformation within the tripeptide (e.g. Ala-X-Ala): and 3) the residue hydrophobicity was obtained from the hydrophobicity scale of Black and Mould [27]. The scale was normalized such that glycine has a hydrophobicity of zero; therefore, amino acids that were more hydrophobic than glycine, were positive and those that were less hydrophobic than glycine were negative on the hydrophobicity scale. The spatial aggregation propensity (SAP) is calculated for spherical regions centred on every atom in the antibody. This gives a unique SAP value for each atom. Then the SAP for a residue is obtained by averaging the SAP of all its constituent atoms.

### *In vitro* analysis

#### Expression of E559 and 62-71-3

The mAbs were expressed as described in our previous study [9]. Briefly, tobacco plants (*Nicotiana benthamiana*: ΔXT/FT plant line) were used as expression system. The chimeric LC and HC gene sequences were cloned into ICON Genetics MagnICON vectors pICH26211 and pICH31160 with TMV and PVX viral backbones respectively. Equal volumes of *Agrobacterium tumefaciens* strain ICF320 (ICON genetics, Germany) cultures containing HC and LC vectors were mixed. The mixtures were diluted to a final OD_600_ of 0.4 for vacuum infiltration. Six weeks old *Nicotiana benthamiana* plants were submerged in the mixed cultures and a vacuum (-800 mbar) was then applied for 3 min. The infiltrated plants were grown at 25°C under a 16 / 8-hour light / dark cycle and harvested after 6 days.

#### Protein extraction and purification

The recombinant mAbs were extracted by homogenizing plants in PBS (15 mM KH_2_PO_4_, 80 mM Na_2_PO_4_H, pH 6.8, 27 mM KCl and 140 mM NaCl) buffer, at a 1:1 ratio, using a blender. The extract was centrifuged (8000 x g) for two cycles at 4°C for 30 min. A 1 ml MabSelect SuRe column (GE Healthcare Life Sciences, Little Chalfont, UK) was used to capture and purify the mAbs at a 1 ml/min flow rate. The column was initially equilibrated with Tris-HCl pH 7.4 for three column volumes (CV). During purification, immobilised protein A bound the Fc region of the antibodies with high affinity at neutral pH (7.4). This was followed by washing of the column for five CVs with Tris-HCl pH 7.4. The antibody was eluted from the column at pH 3.0 (100 mM acetic acid) for 10 CVs into collection tubes that contained 1 M Tris-HCl pH 8. Chromatography was performed using an Akta Avant 150 system (GE Healthcare Life Sciences, Little Chalfont, UK). After the purification step, mAb E559 was buffer exchanged into 10 mM Na_2_HPO_4_ pH 6.8, 150 mM NaCl, 0.01% (w/v) Tween 80, while mAb 62-71-3 was buffer exchanged into 10 mM sodium citrate pH 6.0, 150 mM NaCl 0.01% (w/v), Tween 80 [9]. The protein concentration was determined by using the Bradford assay with bovine gamma globulin standards according to the manufacture’s guidelines (Bio-Rad, California, USA).

#### Accelerated stability studies

Accelerated oxidation studies were performed by exposing E559 and 62-71-3 mAbs to 0.5% hydrogen peroxide (H_2_O_2_) for 4, 20 and 48 hrs at room temperature. After incubation, samples were reduced, alkylated, and digested with chymotrypsin (1:10, enzyme to protein ratio), each time point experiment was conducted in duplicates.

#### In-solution digestion

To unfold the mAbs, a final concentration of 1% SDS (w/v) was added to the mAb samples. Samples were then reduced with 10 mM DTT at 45°C for 45 min and alkylated in the dark with 30 mM iodoacetamide (IAA) for 30 min at room temperature. Sample clean-up and on-bead digestion was performed using MagReSyn HILIC beads (a gift ReSyn Biosciences, Pretoria, South Africa). All experiments were performed with a KingFisher Duo (Thermo Scientific, Massachusetts, USA) magnetic particle processing robot. The automated HILIC-protein clean-up program was developed using BindIt Software 3.0 (Thermo Scientific, Massachusetts, USA) and is available upon request (info@resyinbio.com) to run on any KingFisher Duo system.

The KingFisher Duo system was configured for automated HILIC-protein clean-up and on-bead trypsin digestion. In brief deep-well 96 plates were loaded in each carousel position with each plate filled as follows: 1) 96 well tip heads (Thermo Scientific, Massachusetts, USA); 2) 10 μl, 20 mg/ml hyper porous magnetic HILIC micro spheres (MagReSyn HILIC) in 20% ethanol and 180 μl Equilibration buffer (100 mM NH_4_Ac, 15% acetonitrile (ACN) pH 4.5); 3) Equilibration Buffer (500 μl); 4) Protein extract mixed 1:1 with bind buffer (200 mM NH_4_Ac, 30% ACN pH 4.5), final volume of 100 μl; 5) 500 μl 95% ACN (wash 1); 6) 500 μl 95% ACN (wash 2); 7) 200 μl 50 mM Ammonium Formate pH 8.2 and Promega sequencing grade Trypsin for an enzyme: protein ratio of 1:10.

The Bindit program was then run with the magnetic pins transferring the magnetic HILIC beads from position 2 to 8 and in the process binding proteins, washing off SDS and other contaminants and finally generating peptides ready for LC-MSMS analysis post the on-bead trypsin digest.

#### Reverse phase liquid chromatography electrospray ionization time of flight mass spectrometry

Dried peptide samples were re-suspended in 2% (v/v) ACN / 0.2% (v/v) formic acid (FA) and desalted on an Acclaim PepMap C18 trap (100 μm ID x 2 cm, 5 μm, 100 Å). The peptides were separated on an Acclaim PepMap C18 (300 μm ID x 150 mm, 3 μm, 120 Å) reverse phase column connected via a 10-port switch valve of the Dionex Ultimate 3000 nanoRSLC system (Thermo Scientific, Massachusetts, USA). The peptides were eluted by an ACN gradient (5–35% in 15 min at 8 μl/min) and samples were then electrostatically sprayed in the ESI source and introduced into a 6600 Triple TOF (ABSciex, Massachusetts, USA) operated in Data Dependant Acquisition mode. Precursor MS scans were acquired from *m/z* 400–1500 using an accumulation time of 250 ms followed by 80 MSMS scans, acquired from *m/z* 100–1800 at 25 ms each, for a total scan time of 2.3 sec. Multiply charge ions (2^+^ - 5^+^, 400–1500 *m/z*) were automatically fragmented in Q2 collision cells using nitrogen as the collision gas. Collision energies were chosen automatically as function of *m/z* and charge.

PEAKS Studio version 6 [28] was used to match experimental peptide mass data to the theoretical masses calculated from the amino acid sequences of the mAbs. The parent mass error was set to 25 ppm while the fragment mass error was set to 0.05 Da. A maximum of 3 missed cleavages were allowed for trypsin digestion while a maximum of 4 missed cleavages were set for chymotrypsin. The peptide spectrum matches were reported at 0.1% false discovery rate (FDR) with ≥ 1 unique peptide per protein.

Label free MS1 quantification using Skyline was performed according to [29] and the manufacture’s tutorial guidelines (http://proteome.gs.washington.edu/software/ skyline). Under the Skyline peptide settings tab, chymotrypsin was selected as enzyme and a maximum of 4 missed cleavages were allowed. The time window for the measured retention time was set to 2 min. The minimum peptide length was set to 5 and the maximum length was set to 25 amino acids. A spectral library with a cut-off score of 0.95, was created in the file menu by importing peptide searches based on data dependent acquisition MS/MS data from PEAKS Studio version 8 [28].

#### Circular dichroism

Samples were analysed in their formulation buffers using a Chirascan CD Spectrometer: Applied Photophysics, Leatherhead, UK. A 1 mm cuvette was used. Prior to sample analysis, the buffer interference was tested [30]. All collected spectra were normalised by calculating the mean residue ellipticity [θ] deg.cm^2^dmol^-1^residue^-1^[31].

#### Fluorescence spectroscopy

Fluorescence spectroscopy was carried out in a Shimadzu RF-530K Spectrofluorophotometer (wavelength accuracy of +/- 1.5 nm). Emission spectra of the samples were recorded in the range of 280–450 nm and the excitation and emission slit widths were both set to 5 nm. The excitation wavelength was set to 280 nm for both tryptophan and tyrosine. Tryptophan gives the highest quantum yield (emitted photons) and was therefore selectively excited at a wavelength of 295 nm [32,33].

#### Hydrogen/deuterium exchange mass spectrometry

The effect of F/T was investigated by pulse labelling samples for 15 s after F/T cycle 1, 3, 5 and 7 and before the samples were frozen [28]. One cycle refers to a sample being frozen in liquid nitrogen for 5 min and subsequently thawed at room temperature for 5 min. This allowed for investigation of the temporal sequence of events that lead to formation of unfolded states [34].

Peptide level H/DXMS was conducted using an Agilent HPLC (California, USA) connected to a PALL Leap HDX robot (Leap Technologies, Florida, USA) that had a pepsin column (Applied Biosystems, California, USA), Dionex (Thermo Scientific, Massachusetts, USA) peptide trap (LC packing, ID: 1.0 mm, phase C18PM) and C18 (50 x 2.10 mm, Aeris Peptide 3.6 μm particle size) reverse phase column (Phenomenex, California, USA) installed inside the temperature controlled column compartment which was set to 4°C. Purified samples were prepared under deuterated conditions by 8-fold dilution in D_2_O. The non-deuterated samples were diluted by the same buffer but it contained H_2_O instead of D_2_O. Samples were quenched at 0°C and low pH quenching buffer (50 mM Na_2_HPO_4_, pH 2.5, 0.45 M glycine, 0.625 M tris (2-carboxyethyl) phosphine (TCEP) and 4.2 M CH_6_ClN_3_). The reduced sample was then digested with acid stable immobilised pepsin. This was followed by trap desalting and rapid liquid chromatography separation. The peptides were eluted by an ACN gradient (10–25% B, for 10 min at 200 μl/min). Peptide maps (non-deuterated samples) were generated with MS1 and MS2 as per 2.3.5 and searched on PEAKS Studio version 6 [28]. Protein sequences were imported into HD Examiner (Sierra analytics, California, USA) and analysed according to the manufacture’s guidelines.

To monitor the significance of the changes observed due to F/T, a two-sample t-test was performed using the Perseus software version 1.6.0.2 [35][36]. Duplicate runs were grouped and cycle 0 was compared to F/T cycle 1, 3, 5 and 7. The FDR was set to 1% and the S0 value was set at a default value of 0.1. The S0 value controlled the relative importance of the resulted p-value and difference between means; adjusted p-value cut off was set to 0.05 (results below this value were reported as significant) [35]. The results were shown in a form of a volcano plot.

## Results and discussion

Rabies mostly affects people that reside in remote, resource-limited areas where there are occasional failures in the cold-chain. These environmental changes may upset the finely tuned balance of the non-covalent contacts that stabilise the native conformation of mAbs [34]. *In silico* tools were initially used to identify aggregation prone regions. This was followed by *in vitro* structural analysis of mAbs E559 and 62-71-3 and their response to F/T and exposure to reactive oxygen species, by using several biophysical techniques, such as circular dichroism, fluorescence spectroscopy and deuterium exchange mass spectrometry.

### *In silico* analysis

#### Identification of aggregation prone regions

Stability of proteins has been shown to depend on the packing of their hydrophobic and hydrophilic amino acids [26]. The E559 and 62-71-3 mAbs were therefore modelled (S1 Fig) to calculate the relative solvent accessibility (RSA) of their amino acids and thus help predict regions that may influence their stability. The Fc regions of the chimeric E559 and 62-71-3 were identical and therefore modelled with the same template. Areas that contribute toward E559 relative instability were therefore hypothesised to be in the Fab region.

Before proceeding with further analysis of the modelled structures, their quality had to be evaluated. This was done with the aid of PROCHECK version 3.6.2 and WHAT-CHECK. Ramachandran plot results were presented in table format (S1 Table). The E559 and 62-71-3 structures had a good quality with over 90% of the residues found in the most favoured regions. The second part of the table shows the G-factors which provided a measure of how unusual, or out-of-the-ordinary, different properties were. Values below -0.5 would be unusual and values below -1.0 would be highly unusual. Values of the distribution of main-chain and side-chain dihedral angels, geometry and bonds were all above -0.5, which further illustrates that the E559 and 62-71-3 structures had good quality [24,37].

**Fig 1:**
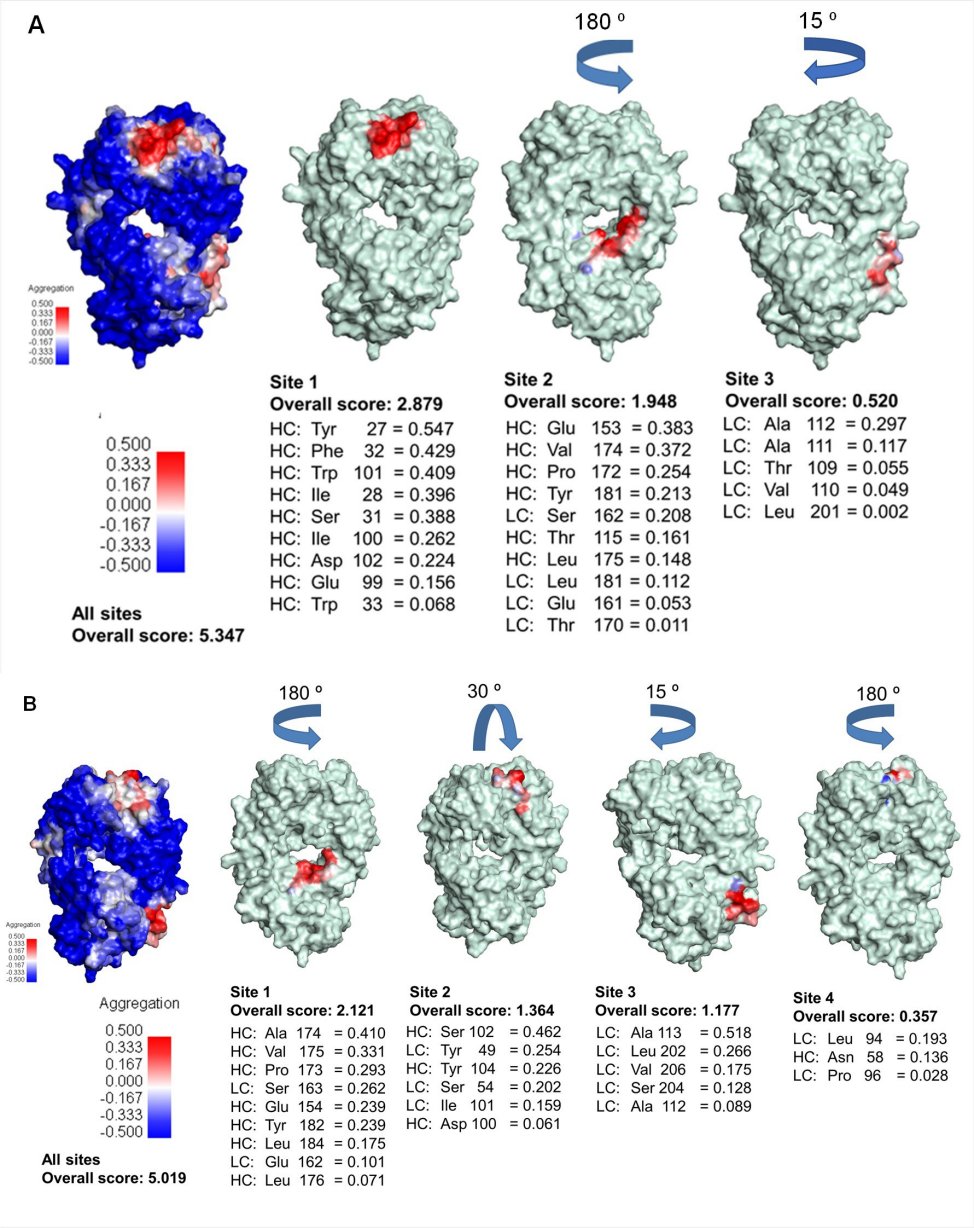
**Spatial aggregation propensity of the Fab region of E559 (A) and 62-71-3 (B) at R = 10 Å.** The SAP values at R = 10 Å were mapped on the Fab fragments. The red patches indicate sites that have a high propensity for aggregation while the blue patches are less prone to aggregation. The APRs were ordered by their contribution to the overall aggregation score.

The SAP tool was used to map aggregation prone regions (APRs) (Fig 1A and 1B). It was selected on the bases that unlike other tools such as Zyggregator, Aggrescan PASTA and SALSA, the 3D structure is used as an input file instead of the primary sequence. This allows for surface exposed hydrophobic patches to be highlighted on the 3D structure that may act as structural hotspots for aggregation. The SAP tool has also been validated by predicting and reducing aggregation propensity of IgG1 mAbs (E559 and 62-71-3 are IgG1 mAbs), by mutating amino acids found in aggregation prone regions and demonstrated that it can be applied to improve development of biotherapeutics [38].

The Fab region of E559 had three APRs (Fig 1A) while that of 62-71-3 had four (Fig 1B). However, the overall aggregation score of E559 (5.347) was higher than that of 62-71-3 (5.019), which possibly indicates that E559 was more prone to aggregation then 62-71-3. The E559 CDR regions contributed the most to the overall aggregation score. These regions were dominated by hydrophobic residues like Tyr27, Phe32, Trp101, Ile28 and Ile33 which highly contributed to the aggregation score compared to Tyr49, Tyr104 and Ile 101 found in the CDR region of 62-71-3. This indicates that E559 is more at risk of losing its efficacy as these hydrophobic residues associate in attempts to escape solvent exposure. However, these predictions were based on one snapshot of the mAb conformation, which may not be the true representation of their behaviour in solution. *In vitro* analysis of the mAbs was therefore performed to confirm these findings.

### *In vitro* analysis

#### Analysis of the mAbs at their primary and secondary structure level

The extracted and purified mAbs were initially verified by approximating their molecular masses using SDS-PAGE (S2 Fig) under reducing conditions that allowed for separation of the HC and LC. The HC migrated to approximately 49 kDa while the LC migrated to approximately 25 kDa which are the correct mass for a typical IgG1 molecule [11]. However, E559 had a double band on the LC which was reported in our previous work to be due to glycosylation [9]. The sequences were verified at their primary level by excising the protein bands as per the protocol described in [39]. Proteins were digested over night at 37 C° using 5–50 μl, 10 ng/μl tryspin depending on the gel piece size this was followed by LC-MS/MS analysis.

The native secondary structures were assessed by using Far-UV CD spectroscopy. The spectra displayed a negative curve with the minimum at 217 nm, which indicated that the structures were dominated by β-sheets (S3 Fig) [40,41]. This was indicative of a typical immunoglobulin fold and also corresponded to the homology models that are dominated by β-sheets.

#### The tertiary structure of the mAbs

**Fig 2:**
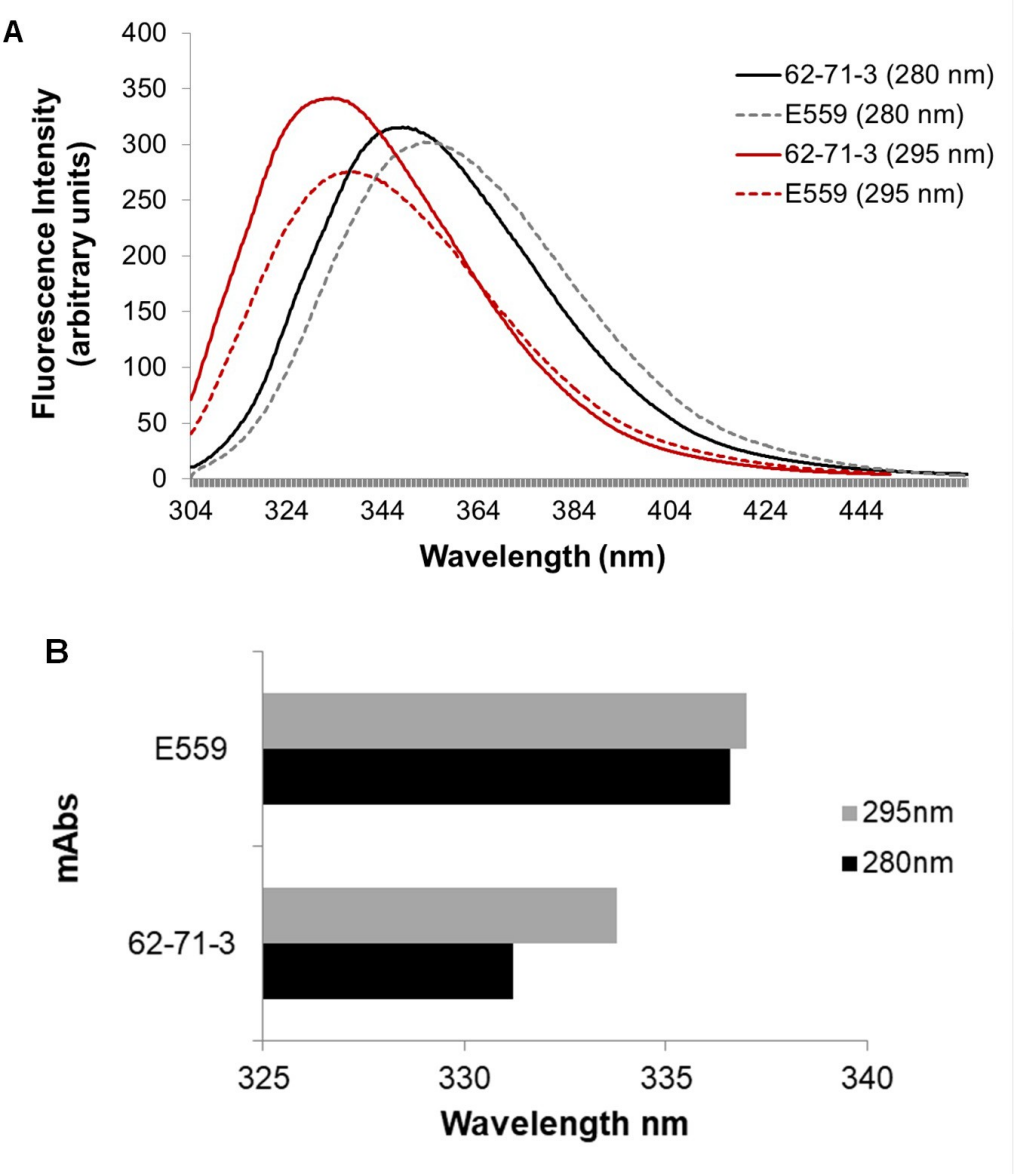
Intrinsic fluorescence emission spectra of native E559 and 62-71-3 excited at 280 nm. Graph A shows the fluorescence emission spectra of E559 (excited at 280 nm: dotted grey line; excited at 295 nm: dotted red line) and 62-71-3 (excited at 280 nm: black solid line; excited at 295 nm: solid red line). The spectra are averages of three accumulations. Graph B illustrates the shift in wavelength between both mAbs. The maximum emission wavelength (280 nm: black bar and 295 nm: grey bar) for 62-71-3 and E559.

Fluorescence spectroscopy was used to compare the tertiary structures of E559 and 62-71-3, by exciting both Trp and Tyr residues at 280 nm and selectively exciting Trp at 295 nm [32,33]. The E559 mAb has a total of 58 Tyr and 24 Trp residues, while the 62-71-3 has 56 Tyr and 22 Trp residues distributed throughout its structure. E559 showed a larger shift towards red for the maximum emission wavelength compared to 62-71-3. This shift was present at both excitation wavelengths (Fig 2). This indicated that E559 had more Trp/Tyr residues exposed to the solvent. The results suggested that the E559 was more loosely packed compared to 62-71-3. This possibly makes E559 more susceptible to aggregation as these hydrophobic residues could promote protein-protein interactions as they try to reduce their contact with water molecules [42]. However, the distribution of Trp and Tyr residues in both mAbs are evenly spread throughout their structure which makes it difficult to determine which region in E559 is responsible for the red shift. The behaviour of both antibodies during F/T was therefore investigated using H/DXMS which allowed us to identify specific regions that are highly prone to degradation.

#### Monitoring the effects of freezing and thawing using H/DXMS

**Fig 3:**
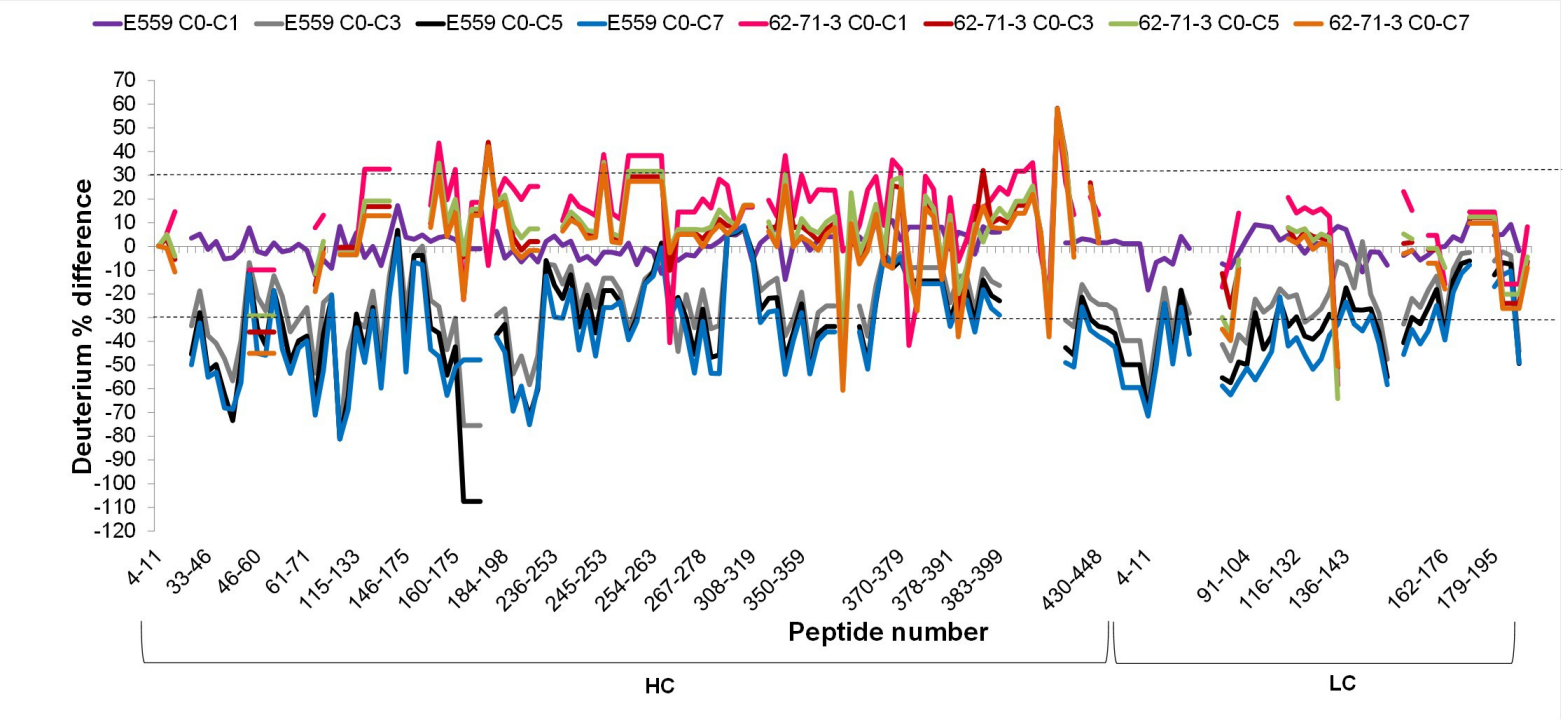
Difference in deuterium incorporation for peptides from E559 and 62-71-3 after F/T cycles 1, 3, 5 and 7. Structural perturbation due to freezing and thawing was monitored by subtracting the percent deuterium exchanged after 15 s on a non-frozen sample (C0) by the percent exchanged after F/T cycle 1, 3, 5 or 7.

The impact of F/T was investigated by using SEC at Kentucky Bioprocessing (S4 Fig). The investigation was carried out over 21 days for up to 3 F/T cycles. The 62-71-3 mAb remained highly stable with only 3% loss in the full mAb population after the third F/T cycle. Aggregation of mAb E559 increased with multiple F/T events, resulting in a loss of 12% of the full mAb population after the third F/T cycle. To determine where these changes occurred, pulse labelling H/DXMS was used to investigate structural perturbations of the mAbs due to F/T by providing an instantaneous measure of the folded/unfolded populations [43]. Cycle 0 represented a non-frozen sample. The changes induced by F/T were determined by subtracting the deuterium percentage of the non-frozen sample with the deuterium percentage after F/T cycle 1, 3, 5 and 7, run in duplicates. E559 was affected more by F/T then 62-71-3 as indicated by the significantly increased deuterium incorporation observed with consecutive F/T cycles as compared to 62-7-13 (Fig 3).

**Fig 4:**
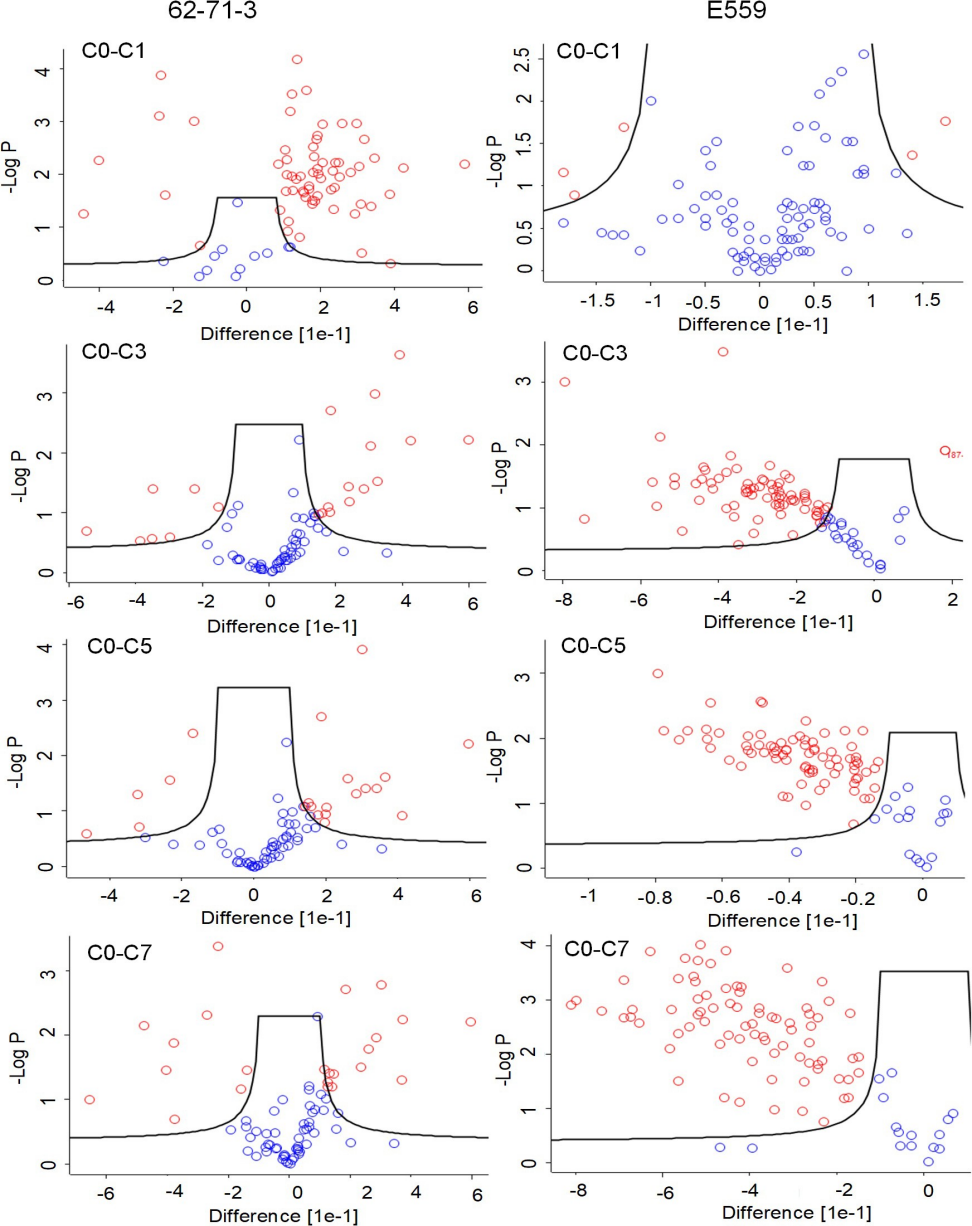
**Volcano plots illustrating the effect of freeze-thawing on 62-71-3 (A) and E559 (B) HC peptides.** The black solid line delineates peptides that show a significant shift in deuterium incorporation (red dots) versus those that were not affected by F/T (blue dots). Samples were analysed in duplicates. C0 represents non-frozen sample, C1, C3, C5 represent F/T cycle 1, 3, 5 or 7. The circles with a negative (-) difference indicate increased deuterium incorporation compared to the non-frozen sample. The circles with a positive (+) difference indicate decreased deuterium incorporation compared to the non-frozen sample. The FDR was set to 1%, S0 value was set to 0.1 and adjusted p-value cut off was 0.05.

**Fig 5:**
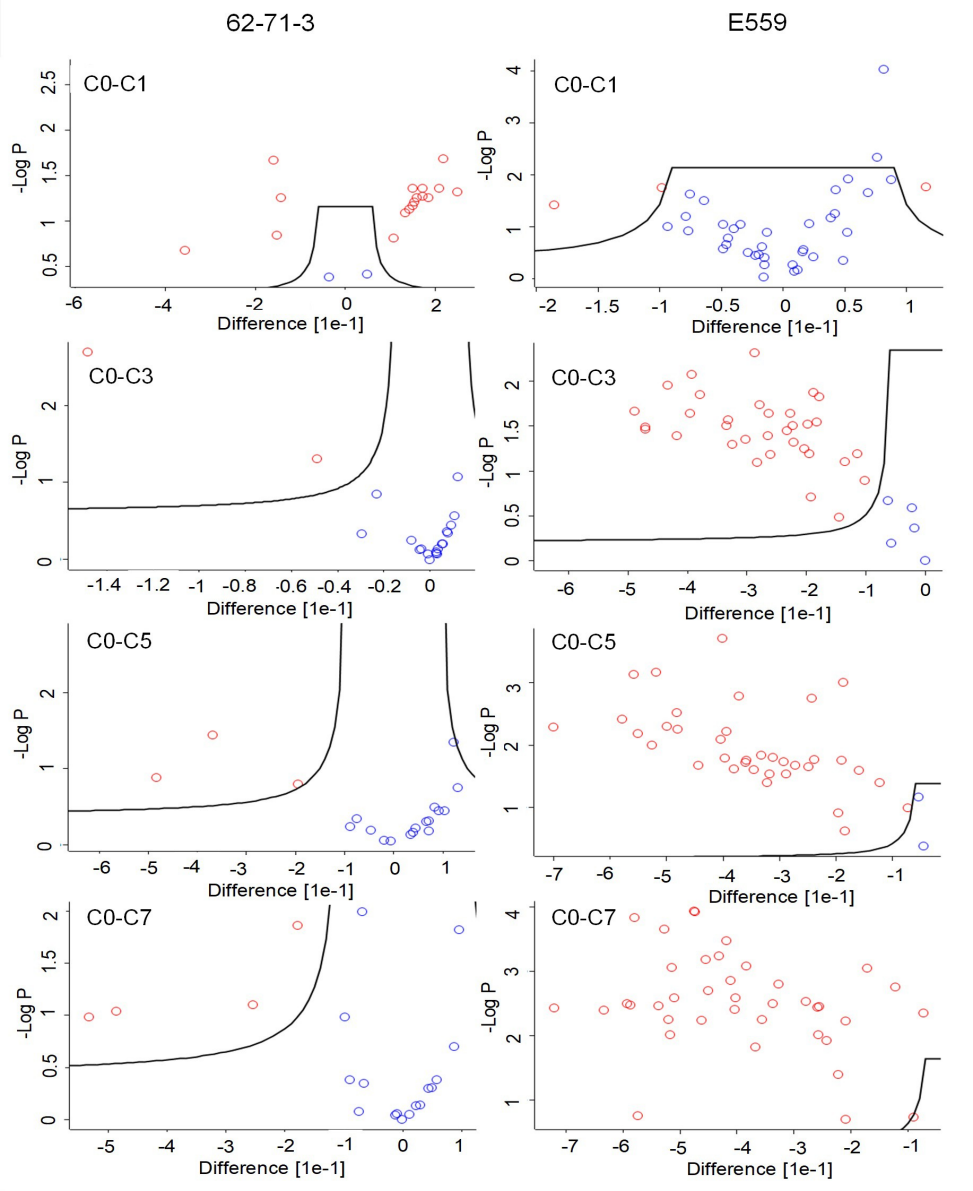
**Volcano plots illustrating the effect of freeze-thawing on 62-71-3 (A) and E559 (B) LC peptides.** The black solid line delineates peptides that show a significant shift in deuterium incorporation (red dots) versus those that were not affected by F/T (blue dots). Samples were analysed in duplicates. C0 represents non-frozen sample, C1, C3, C5 represent F/T cycle 1, 3, 5 or 7. The circles with a negative (-) difference indicate increased deuterium incorporation compared to the non-frozen sample. The circles with a positive (+) difference indicate decreased deuterium incorporation compared to the non-frozen sample. The FDR was set to 1%, S0 value was set to 0.1 and adjusted p-value cut off was 0.05.

To monitor the significance of the changes observed in Fig 3, Runs were grouped and cycle 0 was compared to F/T cycle 1, 3, 5 and 7. The FDR was set to 1% and the S0 value was set at a default value of 0.1, to evaluate the relative importance of the t-test p-value and the differences between the means within the groups [35]. The results were shown in the form of a volcano plot (Figs 4 and 5). The solid line showed the significance cut-offs based on 1% FDR and 0.1 S0 values [35]. Red circles indicated peptides that were significantly different from cycle 0 while blue circles indicated peptides that did not change significantly between F/T cycles.

When evaluating the pattern of significantly changing peptides for 62-71-3 HC (Fig 4A) and LC (Fig 5A), we noted that the largest difference occurred after cycle 1. Most of the peptides were found on the positive side of the difference curve which meant that those peptides had a lower percentage of deuterium uptake, which indicated protection from deuterium incorporation. There were also peptides that had a higher percentage deuterium uptake after F/T cycle 1 which were observed on the negative side. From cycle 3 most of the peptides seemed to go back to their original state, which indicates possible reversible aggregation/oligomerization with a few peptides affected by the F/T.

E559 showed an increased percentage deuterium uptake after cycle 3 which indicated that the mAb started to unfold. This makes the residues more exposed to the surrounding aqueous environment which may eventually induce aggregation as hydrophobic residues try to escape solvent exposure [28]. The peptides that were affected by F/T had amino acids that were predicted by the SAP tool to be prone to aggregation.

#### H/DXMS and *in silico* data correlation

**Fig 6:**
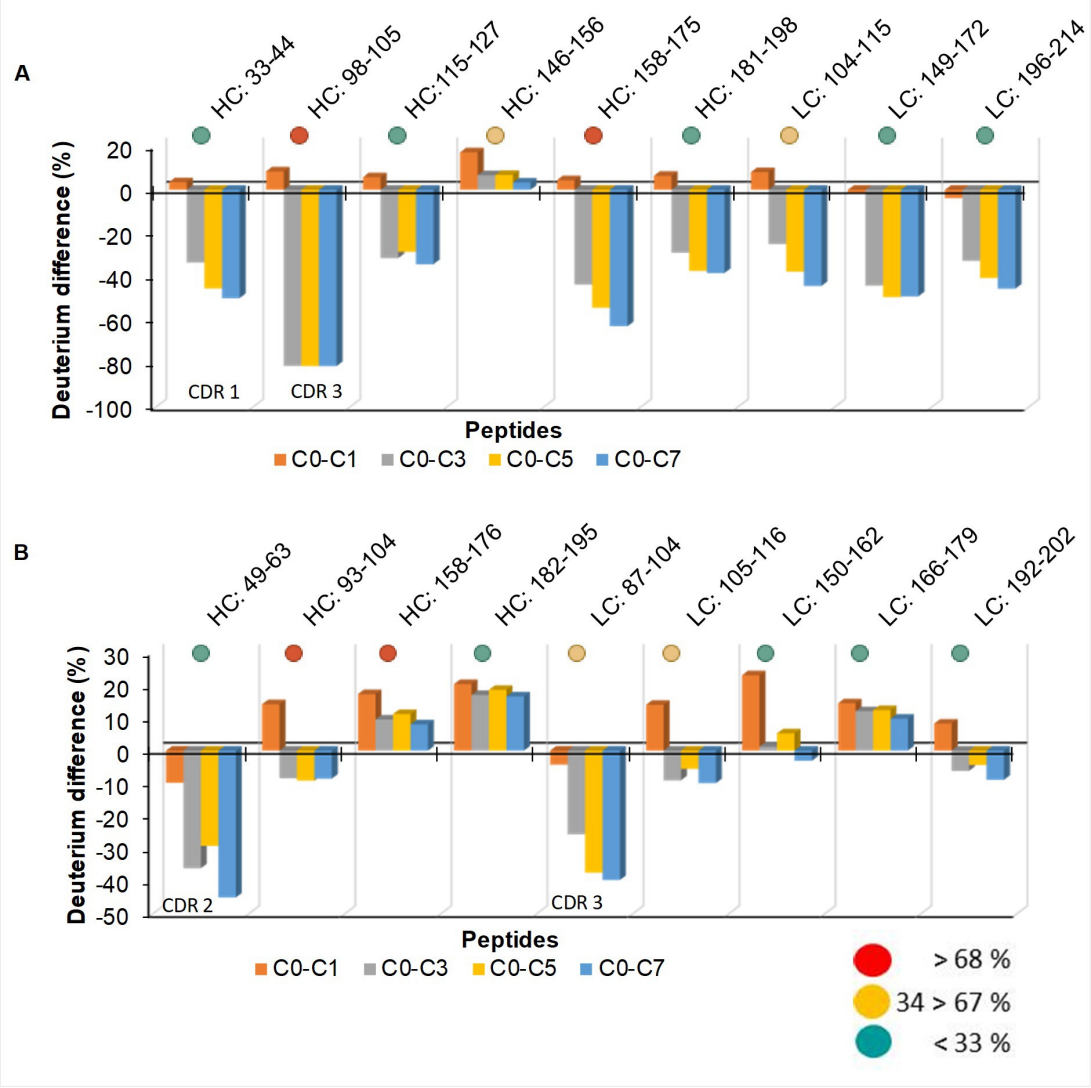
**Differences in deuterium incorporation for E559 (A) and 62-71-3 (B) and their contribution to the SAP aggregation score.** Deuterium incorporation in cycle 0 was deducted from cycle 1, 3, 5 and 7. The contribution of each peptide to the SAP aggregation score was calculated, red circles indicate high contribution, yellow is medium contribution and green is low contribution.

Fig 6 integrates data from SAP prediction (Fig 1) and F/T H/DXMS experiments (Figs 4 and 5). Peptide “^98^R**EIWD**GGF^105^” (residues in bold were identified in aggregation prone regions in Fig 1) which is found in the CDR region (E559 HC) was the most affected by F/T. It was followed by peptide “^158^SWNSGALTGH**T**FPA**VL**^175^” which is located in the constant region of the E559 Fab domain. These results from H/DXMS also correlated with the SAP score (Fig 1A). For the LC of E559, the peptide that contributed the most to the SAP score in site 3 (^104^EIKRTV**AA**PSVF^115^) was also significantly affected by F/T.

Peptides “^181^**Y**S**L**SSVVTVPSSSLGTQT^198^”, “^115^**T**VSSASTKGPSVFPIAPSS^127^” and “^33^**W**MQWARQRRPGQA^44^” were not ideal peptides to be used for comparison as the residues that were detected by SAP to contribute to the aggregation score (marked red) were at the N-terminus where the exchange rate is rapid [44]. There were no other peptides that had these residues positioned away from the N-terminus.

Contradictory to what was computationally predicted for 62-71-3 (Fig 1B), the peptides with the highest percentage deuterium exchange were those found in the CDR regions which was expected since CDRs are typically highly solvent exposed. However, this difference was much lower for 62-71-3 compared to that of E559. This indicated that 62-71-3 had a slower unfolding rate and was therefore more stable than E559.

**Fig 7:**
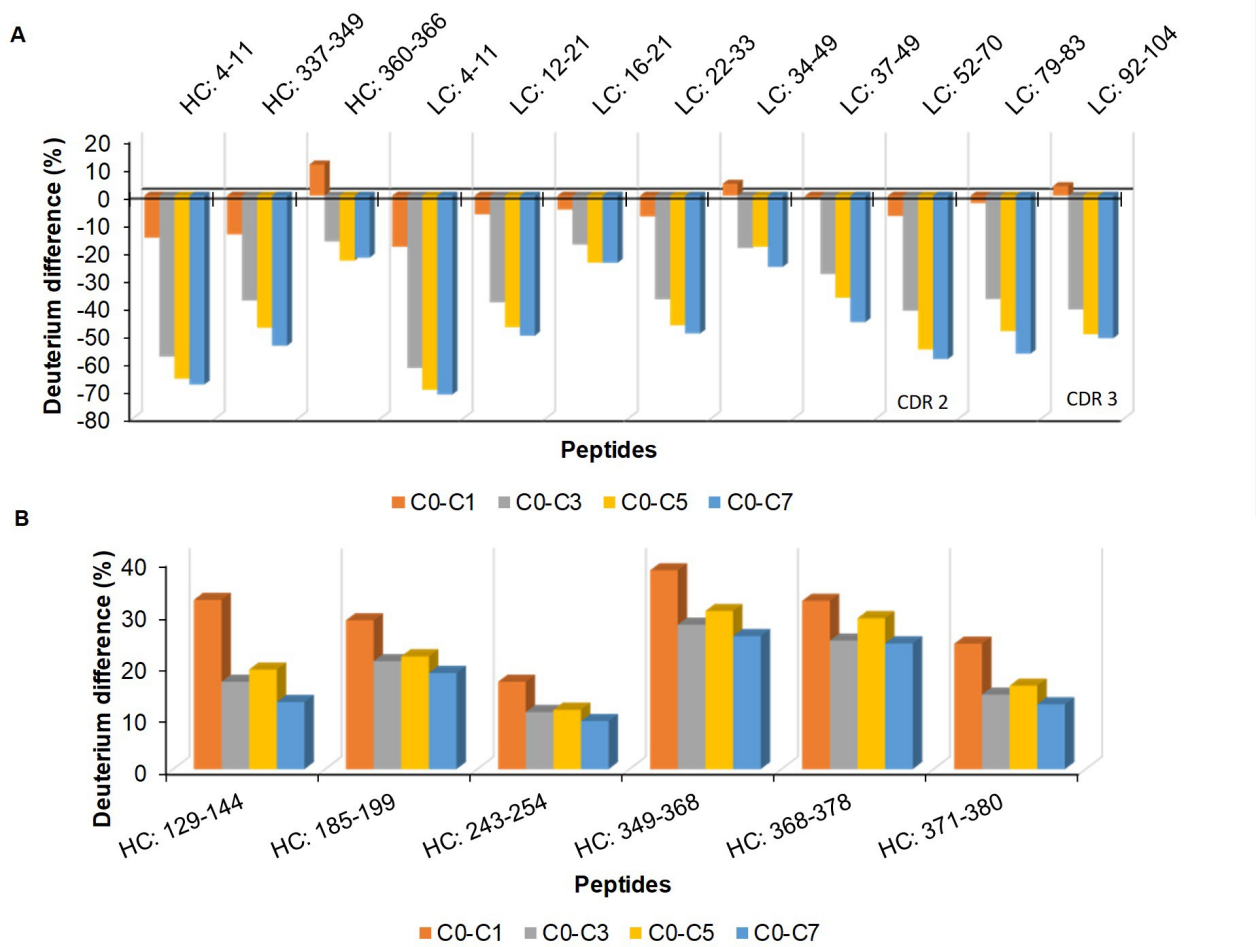
**Differences of deuterium incorporation for E559 (A) and 62-71-3 (B).** Deuterium incorporation in cycle 0 was deducted from cycle 1 (orange), 3 (grey), 5 (yellow) and 7 (blue).

There were also other peptides besides those identified by SAP that were significantly perturbed by F/T in all the cycles (Fig 7). Peptide “^52^**F**SLDSGVPKRFSGSRSGS^70^” and “^92^**ATSPYT**FGGGTKL^104^” from E559 had residues (red) that were found in CDR regions. The CDR regions of E559 were more affected by F/T, thus making E559 more prone to loss of efficacy then 62-71-3.

**Fig 8:**
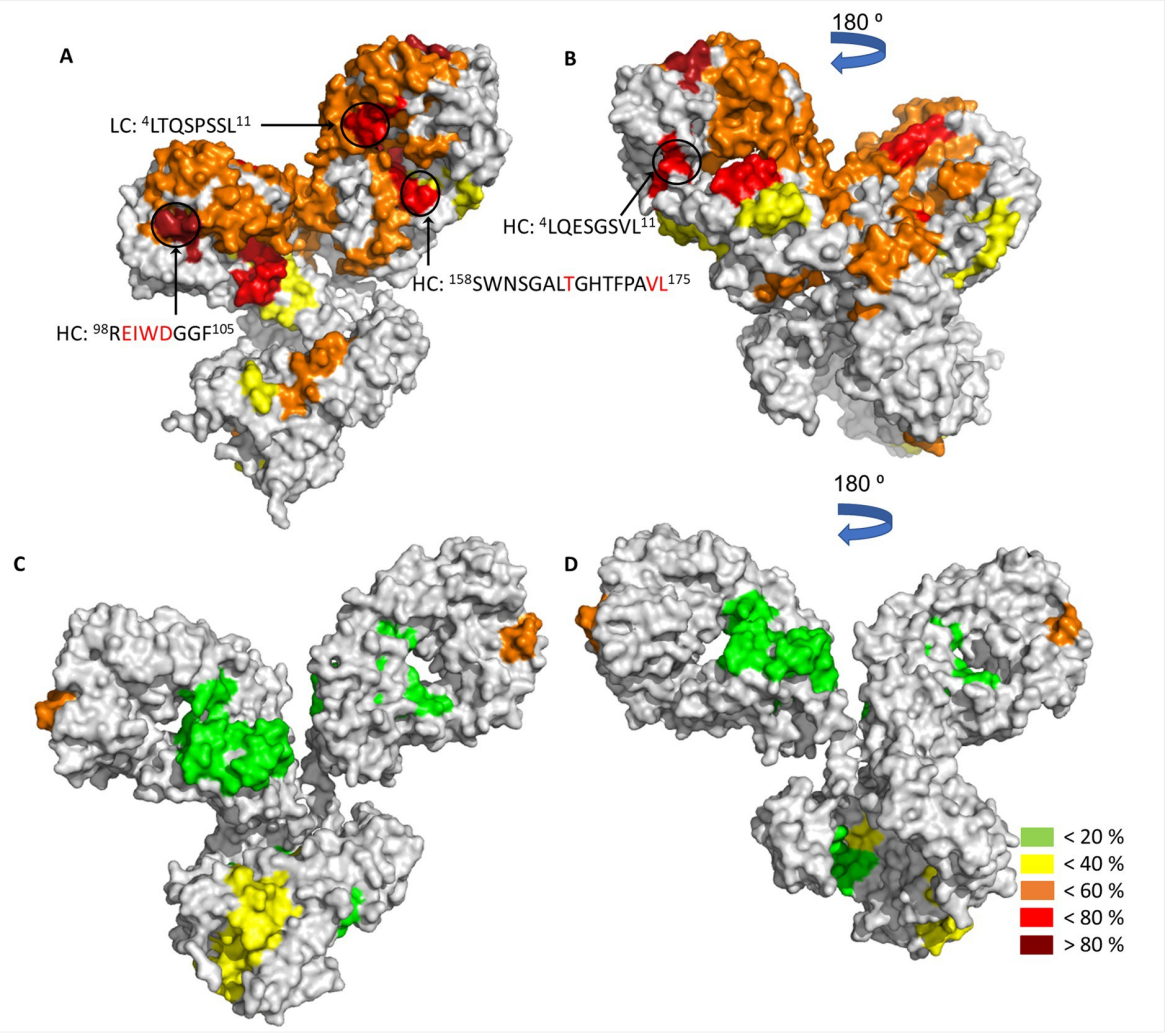
**Peptides that were significantly affected by F/T after cycle 7 mapped on models of mAbs E559 (A and B) and 62-71-3 (C and D).** The difference in deuterium incorporation between the non-frozen cycle and F/T cycle 7 peptides were mapped on the mAb structures. Different colours represent the difference in deuterium incorporation, green indicates lowest deuterium percentage (<20%) and dark red represents the highest deuterium percentage (>80%). E559 peptides (^98^REIWDGGF^105^, “^4^LQESGSVL^11^, ^4^LTQSPSSL^11^ and ^158^SWNSGALTGHTFPAVL^175^) that were impacted the most by F/T were mapped on the structure.

The deuterium incorporation difference of peptides that were significantly affected by freezing and thawing after cycle 7 were mapped on the three dimensional structures of the mAbs (Fig 8). The E559 and 62-71-3 mAbs are chimeric with identical constant regions. However, the E559 had more peptides in the constant region that were unfolded by F/T. This indicates to the amino acid sequence of the variable region as the fundamental reason why E559 was more prone to aggregation. These differences resulted in subtle structural differences that made E559 more flexible and more prone to degradation. E559 had four peptides (three in the variable region: ^98^R**EIWD**GGF^105^, “^4^LQESGSVL^11^, ^4^LTQSPSSL^11^ and one in the constant region ^158^SWNSGALTGH**T**FPA**VL**^175^) that were most impacted by F/T. Except for peptide “^98^R**EIWD**GGF^105^” found in the CDR region, the other peptides may serve as good candidates for mutation to enhance the stability of E559.

#### Oxidation

When it comes to biopharmaceuticals, oxidation is one of the most problematic chemical degradation pathways. Oxidative changes to the protein may increase the susceptibility of the protein by the creation of sticky patches on the surface that encourage formation of unwanted covalent bonds [10]. Forced degradation studies were therefore conducted in duplicates to identify residues that were most susceptible to this modification. Reverse phase LC-MS/MS was used to identify and quantify affected peptides. MS1 peak areas were used as a measure of peptide abundance. As a control, a sample that was freshly thawed and digested but not exposed to H_2_O_2_ was used. This control sample was analysed to identify possible modifications introduced during sample processing.

**Fig 9:**
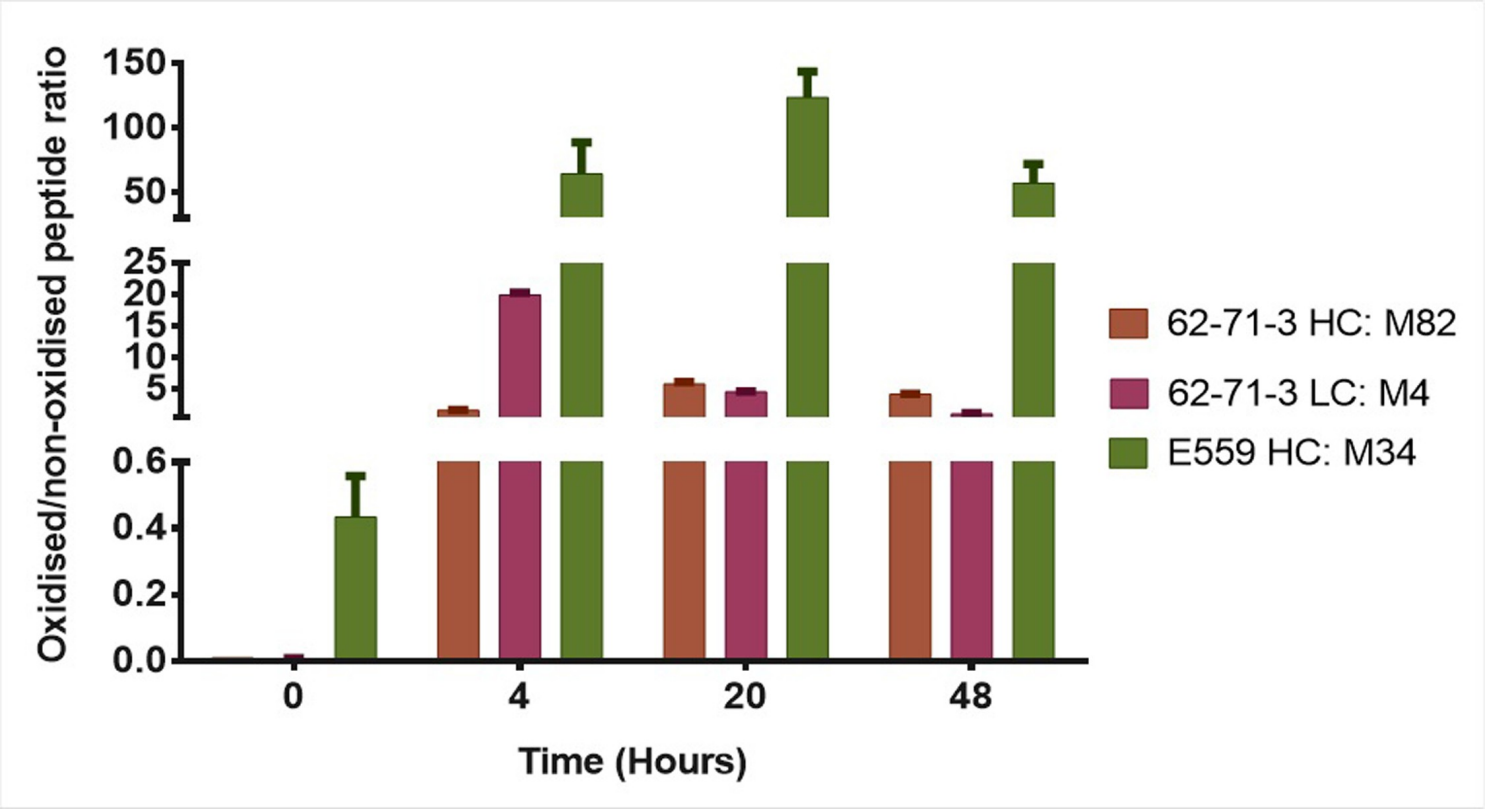
Oxidation levels of 62-71-3 and E559 peptides. The peak areas of oxidised peptides were divided by the peak areas of the un-modified version of the peptide, for duplicate samples incubated for 4, 20 and 48 hrs in 0.5% H_2_O_2_. The control or point 0 was a freshly thawed and digested sample and was included to identify modifications that were introduced during sample preparation.

Skyline software was used to quantify the peptides by measuring their XIC peak areas. Oxidised and non-oxidised peptides were compared within each sample run. The ratios were calculated by dividing oxidised XIC peak areas with their non-oxidised versions. The standard deviations between the duplicate runs were shown in a form of error bars in Fig 9. Data was filtered based on the quality of the spectra, retention time and the isotope dot product (idotp) value. The control samples had low to undetectable oxidised peptides (S1–S4 Files). The opposite was observed for the samples that were exposed to H_2_O_2_ where there was a low abundance of unmodified peptides_._ The idotp value provided a correlation between the expected and observed precursor isotope distribution and the optimal idotp value is 1. Low values were an indication of unreliable isotope patterns, selection of the wrong peak or the signal that was below the detection limit [29].

Met residues were mostly affected by oxidative stress for both mAbs (Fig 9). For 62-71-3, Met82 located in the HC and Met4 located in the LC were highly oxidised. Oxidation of the E559 Met34 was significantly higher than the 62-71-3 Met82 and Met4. Met34 also formed part of the “^33^WMQWARQRRPGQA^44^” peptide that was affected by F/T by becoming more exposed to the solvent with the increase in F/T cycles. It is also next to Trp33 which was predicted by SAP (Fig 1) to be prone to aggregation. Its flexibility and increased exposure to the surrounding aqueous environment may promote aggregation [10].

## Conclusion

The E559 mAb was observed to be less stable than mAb 62-71-3. The CDR regions of E559 are mostly responsible for its instability, more specifically residues ^99^EIWD^102^ found in HC CDR3 and ^92^ATSPYT^97^ found on LC CDR3. The aggregation prone Trp101 from CDR3 was in spatial proximity to Trp33 which formed part of peptides that became more exposed to the solvent with an increase in F/T cycles. These residues were therefore suspected to eventually contribute to unwanted protein-protein interactions via hydrophobic or aromatic interactions. However, mutation of these residues is not recommended as they are found in the CDR region and may play a critical role in binding mAbs to their antigen. The mutation of the E559 Val174 from peptide “^158^SWNSGALTGHTFPAVL^175^” is recommended for future work as it is found in the peptide that was most affected by F/T and it had a high aggregation score. Even though according to the SAP data the E559 peptides “^4^LQESGSVL^11^ and ^4^LTQSPSSL^11^ did not have residues that were prone to aggregation, these peptides were highly affected by F/T. These residues may serve as good candidates for mutation, in the aim to bring forward more stable therapeutic antibodies. Furthermore, Met34 was identified to be highly prone to oxidation; it was also part of a peptide that was significantly affected by F/T, thus increasing susceptibility of E559 to aggregation. Addition of antioxidants in the E559 processing solution may help protect it from oxidation [10].

## Supporting information

S1 Fig Computationally modelled Fab regions of E559 (A) and 62-71-3 (B) mAb and the Fc region (C). One unit of the E559 (A) and 62-71-3 (B) FAB region and Fc dimer region (C) for both antibodies was modelled. The β-sheets were coloured in yellow, helices were coloured red and the coils are in green. The schematic mAb illustrates how the individual regions assemble.
(TIF)

S2 Fig SDS-PAGE showing mAbs after protein A purification. PageRuler Prestained Protein ladder that indicated molecular masses in kDa, was loaded in lane 1. The mAbs E559 and the 62-71-3 were loaded in lane 2 and 3, respectively.
(TIF)

S3 Fig Far-UV CD spectra of E559 and 62-71-3 mAbs. Far-UV CD spectra of E559 (grey dotted line) and 62-71-3 (black solid line) at a concentration of 2 μM. E559 had a total of 1324 residues while 62-71-3 had 1328 residues. Readings were taken in a 1 mm cuvette at 20°C.
(TIF)

S4 Fig Effect of freeze-thawing on the stability profile of E559 and 62-71-3. The full (blue) molecular mass for E559 is 145.5 kDa and 145.4 kDa for 62-71-3. LMM (red) indicate sizes lower than the full mAb while HMM (green) indicates sizes higher than the full mAb. LMM (low molecular mass), HMM (high molecular mass).
(TIF)

S5 Fig Averages of the deuterium percentages and standard deviation for E559 and 62-71-3. Evaluation of the standard deviation between the deuterium up take for the E559 (A) plus 62-71-3 (B) peptides that were plotted in Fig 6 and E559 (C) plus 62-71-3 (D) peptides that were plotted in Fig 7, for Cycle 0,1,3,5 and 7.
(TIF)

S1 Table Ramachandran results for E559 and 62-71-3 mAb Fab and Fc regions. Ramachandran plot statistics and G-factor parameters
(TIF)

S1 File Supplimentary information. 62-71-3 peptide list, oxidation time points and total area.
(CSV)

S2 File Supplimentary information. E559 peptide list, oxidation time points and total area.
(CSV)

S3 File Supplimentary information_62-71-3. 62-71-3 oxidation spectral biospec library.
(BLIB)

S4 File Supplimentary information_E559. E559 oxidation spectral biospec library.
(BLIB)

## Abbreviations

CDR: complementarity determining region
DTT: Dithiothreitol
ERIG: Equine rabies immunoglobulin
F/T: Freeze/thaw
HRIG: Human rabies immunoglobulin
H/DXMS: Hydrogen/Deuterium exchange mass spectrometry
IAA: Iodoacetamide
mAbs: monoclonal antibodies
PEP: Post exposure prophylaxis
RIG: Rabies immunoglobulin
SAP: special aggregation propensity
