# Supplementary figures and images for: The study of degradation mechanisms of glyco-engineered plant produced anti-rabies monoclonal antibodies E559 and 62-71-3
Source: PLoS One. 2018 Dec 20;13(12):e0209373. doi: 10.1371/journal.pone.0209373 (PMC6301680; doi:10.1371/journal.pone.0209373)

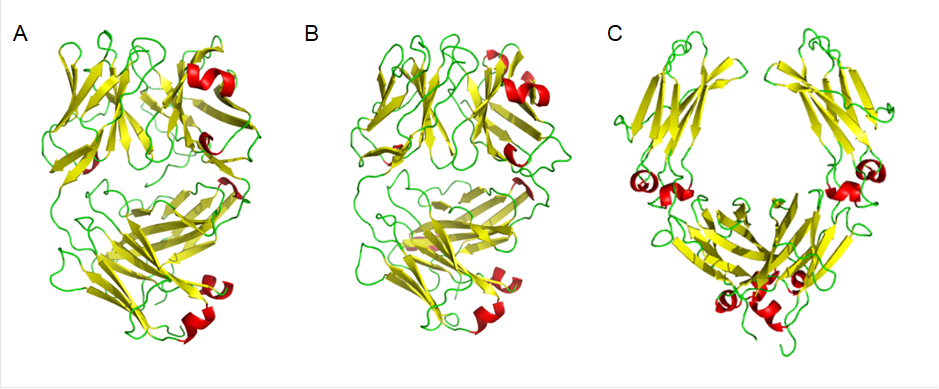

Supplement: S1 Fig — Computationally modelled Fab regions of E559 (A) and 62-71-3 (B) mAb and the Fc region (C). One unit of the E559 (A) and 62-71-3 (B) FAB region and Fc dimer region (C) for both antibodies was modelled. The β-sheets were coloured in yellow, helices were coloured red and the coils are in green. The schematic mAb illustrates how the individual regions assemble. (TIF) [file pone.0209373.s001.tif]

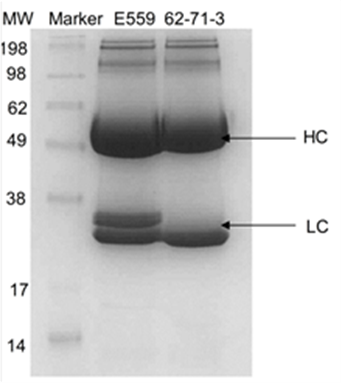

Supplement: S2 Fig — PageRuler Prestained Protein ladder that indicated molecular masses in kDa, was loaded in lane 1. The mAbs E559 and the 62-71-3 were loaded in lane 2 and 3, respectively. (TIF) [file pone.0209373.s002.tif]

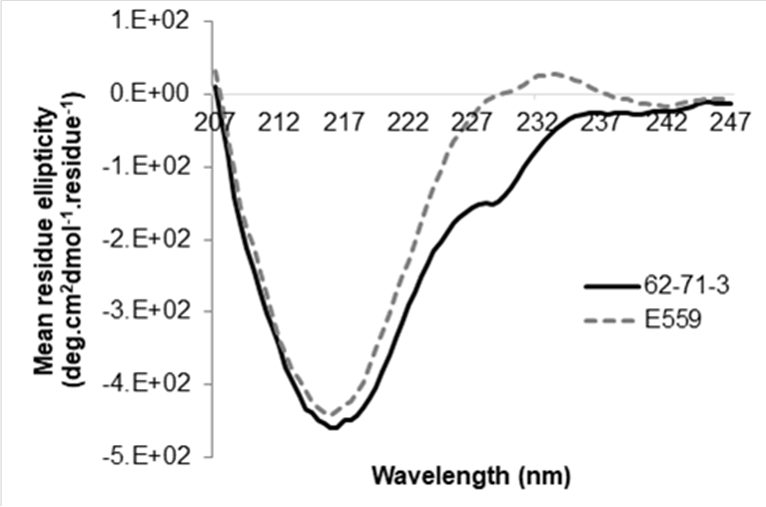

Supplement: S3 Fig — Far-UV CD spectra of E559 (grey dotted line) and 62-71-3 (black solid line) at a concentration of 2 μM. E559 had a total of 1324 residues while 62-71-3 had 1328 residues. Readings were taken in a 1 mm cuvette at 20°C. (TIF) [file pone.0209373.s003.tif]

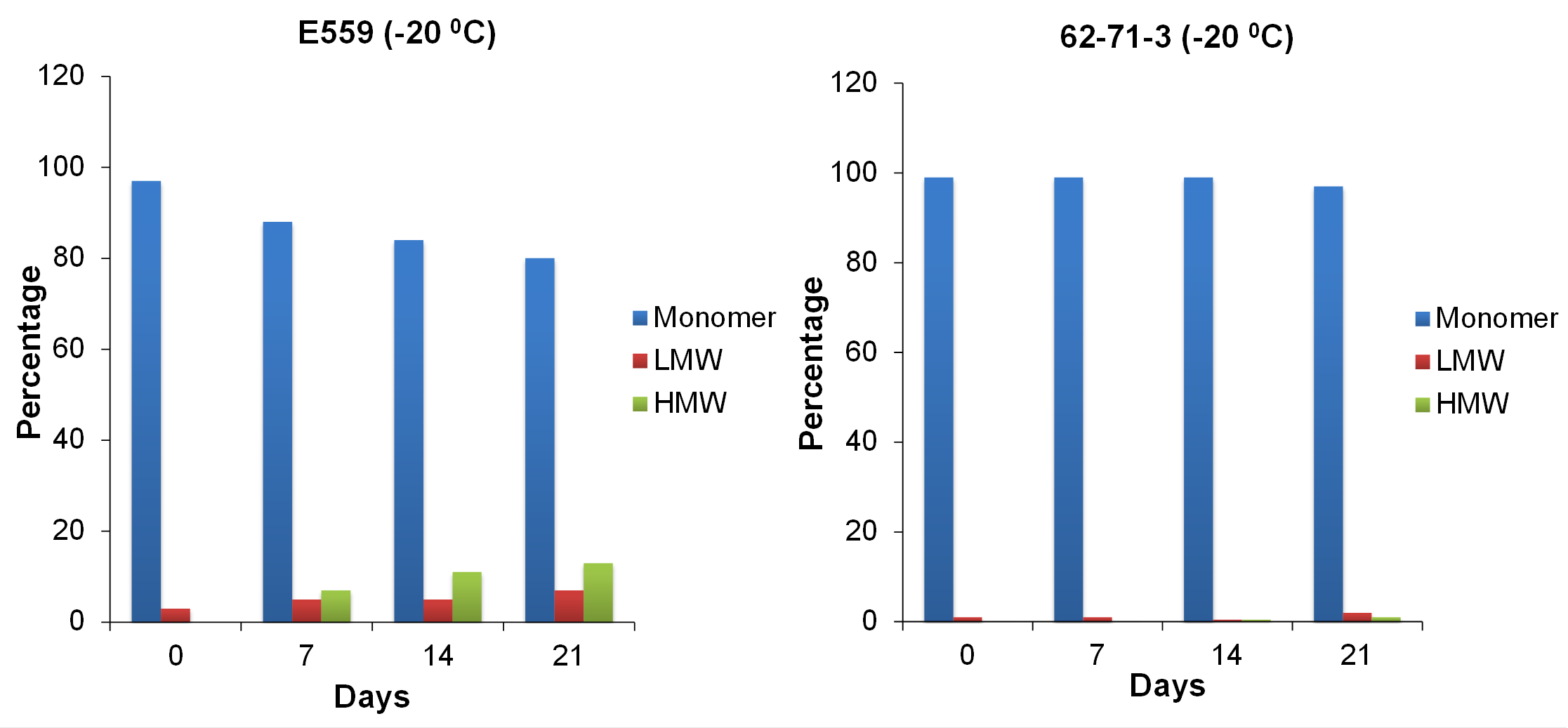

Supplement: S4 Fig — The full (blue) molecular mass for E559 is 145.5 kDa and 145.4 kDa for 62-71-3. LMM (red) indicate sizes lower than the full mAb while HMM (green) indicates sizes higher than the full mAb. LMM (low molecular mass), HMM (high molecular mass). (TIF) [file pone.0209373.s004.tif]

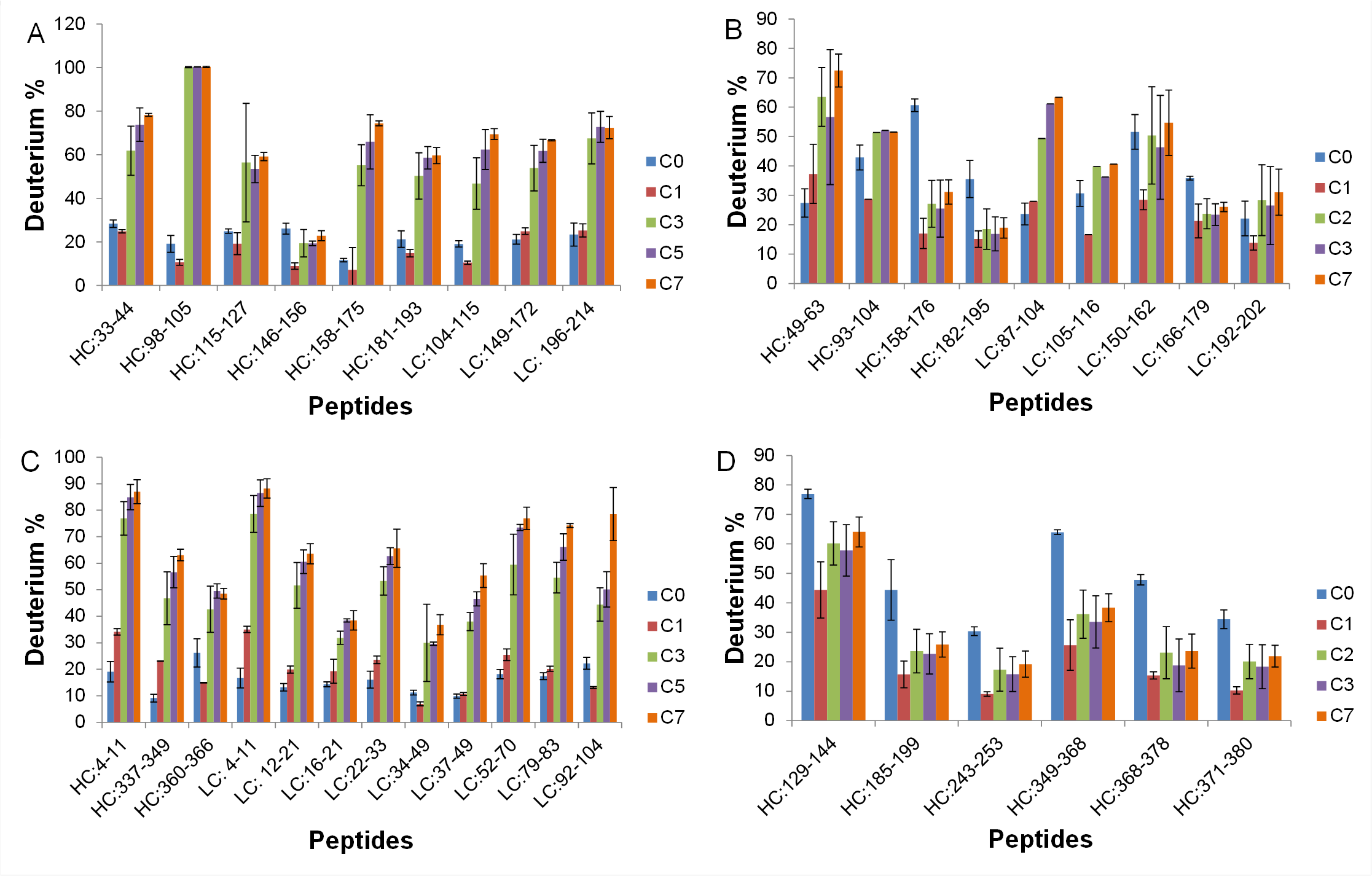

Supplement: S5 Fig — Evaluation of the standard deviation between the deuterium up take for the E559 (A) plus 62-71-3 (B) peptides that were plotted in Fig 6 and E559 (C) plus 62-71-3 (D) peptides that were plotted in Fig 7, for Cycle 0,1,3,5 and 7. (TIF) [file pone.0209373.s005.tif]

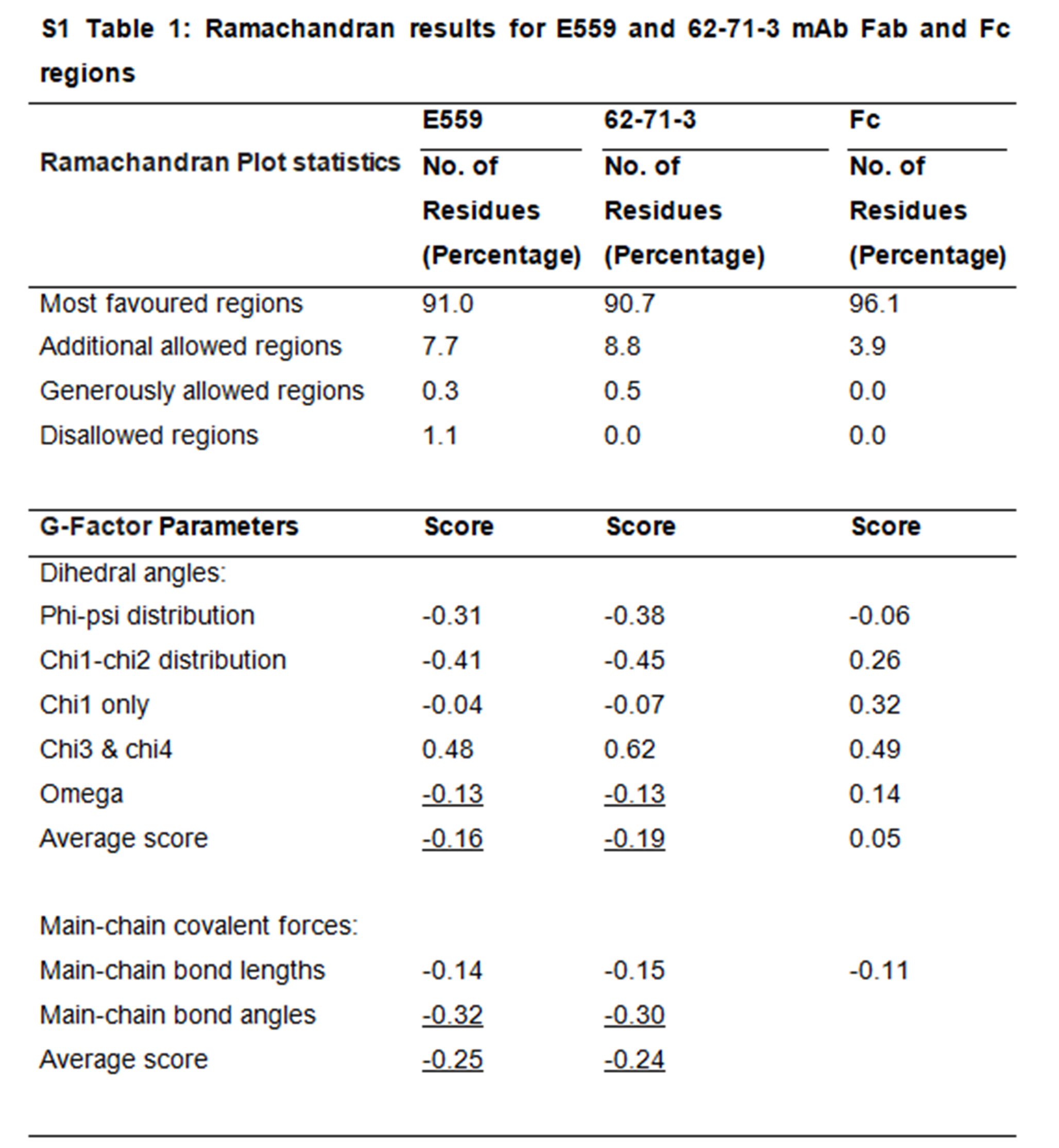

Supplement: S1 Table — Ramachandran plot statistics and G-factor parameters (TIF) [file pone.0209373.s006.tif]
